# Proton minibeam radiation therapy spares normal rat brain: Long-Term Clinical, Radiological and Histopathological Analysis

**DOI:** 10.1038/s41598-017-14786-y

**Published:** 2017-10-31

**Authors:** Yolanda Prezado, Gregory Jouvion, David Hardy, Annalisa Patriarca, Catherine Nauraye, Judith Bergs, Wilfredo González, Consuelo Guardiola, Marjorie Juchaux, Dalila Labiod, Remi Dendale, Laurène Jourdain, Catherine Sebrie, Frederic Pouzoulet

**Affiliations:** 1 Laboratoire d’Imagerie et Modélisation en Neurobiologie et Cancérologie (IMNC), Centre National de la Recherche Scientifique (CNRS); Universités Paris 11 and Paris 7, Campus d’Orsay, 91405 Orsay, France; 2 Institut Pasteur, Histopathologie Humaine et Modèles Animaux, Institut Pasteur, 28 Rue du Docteur Roux, 75015 Paris, France; 3 Institut Curie - Centre de Protonthérapie d’Orsay, Campus Universitaire, Bât. 101, Orsay, 91898 France; 4 Institut Curie, PSL Research University, Translational Research Department, Experimental Radiotherapy Platform, Orsay, France; 5 Paris Sud University, Paris -Saclay University, 91405 Orsay, France; 6 Imagerie par Résonance Magnétique Médicale et Multi-modalités (IR4M-UMR8081), Université Paris Sud, 91405 Orsay, France

## Abstract

Proton minibeam radiation therapy (pMBRT) is a novel strategy for minimizing normal tissue damage resulting from radiotherapy treatments. This strategy partners the inherent advantages of protons for radiotherapy with the gain in normal tissue preservation observed upon irradiation with narrow, spatially fractionated beams. In this study, whole brains (excluding the olfactory bulb) of Fischer 344 rats (n = 16) were irradiated at the Orsay Proton Therapy Center. Half of the animals received standard proton irradiation, while the other half were irradiated with pMBRT at the same average dose (25 Gy in one fraction). The animals were followed-up for 6 months. A magnetic resonance imaging (MRI) study using a 7-T small-animal MRI scanner was performed along with a histological analysis. Rats treated with conventional proton irradiation exhibited severe moist desquamation, permanent epilation and substantial brain damage. In contrast, rats in the pMBRT group exhibited no skin damage, reversible epilation and significantly reduced brain damage; some brain damage was observed in only one out of the eight irradiated rats. These results demonstrate that pMBRT leads to an increase in normal tissue resistance. This net gain in normal tissue sparing can lead to the efficient treatment of very radio-resistant tumours, which are currently mostly treated palliatively.

## Introduction

Proton minibeam radiation therapy (pMBRT) is a novel radiotherapy (RT) strategy recently proposed^1^ in order to lower the main barrier of RT treatments: the tolerance of normal tissues. This approach partners the more precise ballistics of protons with the advantages of minibeam radiation therapy (MBRT). MBRT, originating from synchrotrons^2–4^, combines spatial dose fractionation with the utilization of submillimetre field sizes: the irradiation is performed using an array (“comb”) of thin, parallel beams (“teeth”). The dose profiles in MBRT consist of peaks and valleys, making the peak-to-valley-dose ratio a very important dosimetric parameter^5^. This distinct dose deposition leads to a significant increase in normal tissue tolerance^3,6,7^ compared with standard RT. The classical paradigm of conventional RT in which direct effects of ionizing radiation result in cell death is challenged by the tissue responses to MBRT irradiation, which are influenced by different biological mechanisms and are not yet completely understood. The so-called dose-volume effects^8^, as well as some other contributors, such as cohort effects^9,10^ and prompt vascular repair^9,11^, may play a role.

pMBRT offers several advantages over synchrotron MBRT. First, a negligible dose is deposited in normal tissue after the Bragg peak (tumour position), further reducing the secondary effects. In addition, the multiple coulomb scattering of protons allows the attainment of a homogeneous dose distribution in the tumour with only one array of proton minibeams^1^. In contrast, X-ray MBRT requires two orthogonal arrays, which lead to a more complex and more error-prone irradiation geometry. Moreover, recent studies have demonstrated distinct biological properties of protons^12^, which are not observed or are less frequently observed with photons, such as a complex DNA damage-inducing capacity and an ability to modulate inflammation^12^. These observations support an increase in the therapeutic index of pMBRT over that of X-ray MBRT.

A first proof of the physical feasibility of the technique was performed by Dilmanian *et al*.^13^. In parallel to this work, we have performed the first worldwide technical implementation of this new strategy^14^ at a clinical centre, the Orsay Proton Therapy Center (ICPO-Orsay). For that purpose, a mechanical collimator was employed for minibeam generation^14,15^. A complete set of experimental dosimetric data in such small proton beams was obtained and used to guide the first biological experiments^14^.

The first biological indication of normal tissue (skin) sparing upon the combination of spatial dose fractionation with protons was provided by a recent experiment wherein mice ears were irradiated with 0.18 × 0.18-mm^2^ grid patterns at a research facility^16^. Although neither the beam energy (20 MeV) nor the grid size are clinically relevant^17^, this work provided an initial indication of the potential gain in normal tissue resistance that can be achieved using these approaches.

Our strategy is especially suitable for currently difficult-to-treat neurological indications, i.e., benign and malignant tumours of the brain and spinal cord, head-and-neck tumours, and tumours of the spinal column and the extremities. These are tumours for which pulmonary and/or cardiac cycles have minimal effects. Importantly, the reduced penumbras also make pMBRT a promising treatment for non-cancerous diseases, such as trigeminal neuralgia and epilepsy^18^. Our main target are high-grade gliomas, that are the most aggressive and common brain tumors^19^. To investigate the potential use of pMBRT in future clinical trials, which is our next goal, we aimed to assess the gain in brain tissue sparing via pMBRT compared with conventional proton irradiation in a clinically relevant setting.

## Materials and Methods

### Ethics statement

All animal experiments were conducted in accordance with the animal welfare and ethical guidelines of our institutions. They were approved by the Ethics Committee of the Institut Curie and French Ministry of Research (permit no. 6361–201608101234488). Rats were anaesthetised with isoflurane (2.5% in air) during irradiation and magnetic resonance imaging (MRI). At the end of the study, the rats were terminally anaesthetised for brain fixation by the intracardiac perfusion of formalin zinc.

### Irradiations

The irradiations were performed at one of the horizontal beamlines (passive scattering) at the ICPO-Orsay with a proton beam energy of 100 MeV. This energy allows the treatment of a tumour located at the centre of the human brain (i.e., the worst scenario). This setup enabled the lateral irradiation of rat brains in the plateau region (prescribed dose at a 1-cm depth). The dose rate was 2 Gy/min at a 1-cm depth. The goal was to mimic the irradiation of normal tissues in the clinical treatment of a brain tumour by proton therapy.

**Figure 1:**
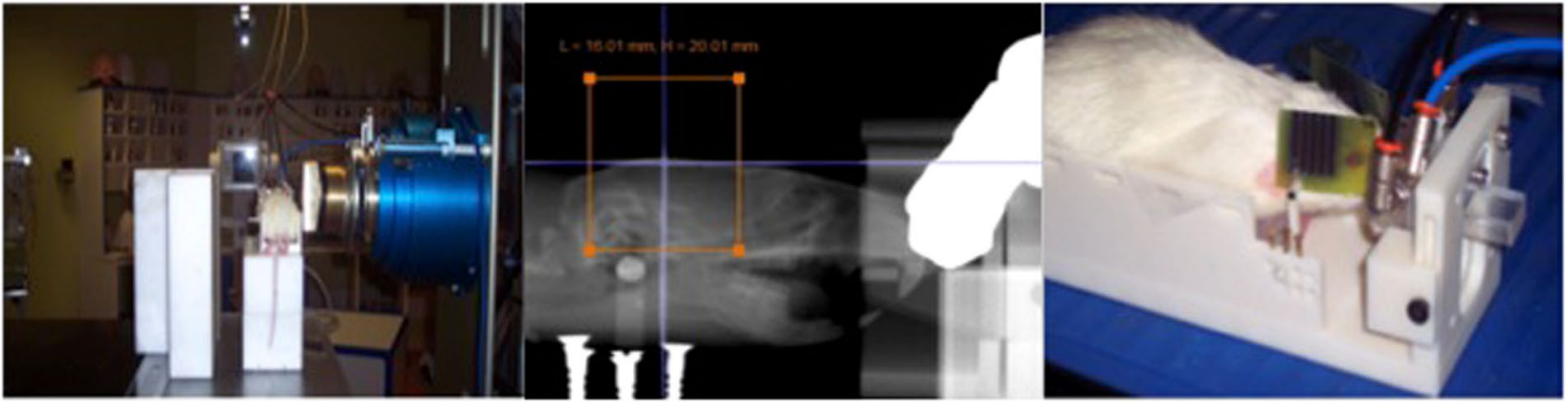
Experimental setup for rat irradiation (left). Photograph of the Gafchromic films used for quality assurance (right). The orange square represents the irradiation field.

Three groups of animals were considered: one group (series 1) receiving conventional (broad beam) proton irradiations (n = 8), a second group (series 2) irradiated with pMBRT, (n = 8) and a control group (series 3, n = 7). The positioning of the rats with respect to the beam was aided by a digital X-ray imaging system (Varian PaxScan 4030E). Anatomical markers were used to define the target region. Special care was taken to avoid the irradiation of the throat. Gafchromic films placed laterally on each side of the rat’s head (beam entry and exit) and attached to the skin allowed assessment of the quality of the irradiation. The rat brains, excluding the olfactory bulb, were irradiated unilaterally (from right to left) with a field size of 1.6 × 2 cm^2^. Figure 1 shows a photograph of the experimental setup (left) and the films used for quality assurance (right). The minibeam patterns are visible in the film.

The same average dose at a 1-cm depth was deposited in the two irradiated series (25 ± 1 Gy). In the case of pMBRT, this corresponds to a peak dose of 57 ± 3 Gy. The dose was delivered in one single fraction to avoid any possible blurring inter-fraction of the minibeam pattern due to positioning errors. As explained in the introduction, our main target are gliomas. Radiation doses higher than 20–25 Gy are reported to be needed to obtain long-term survivals in glioma-bearing rats experiments^20,21^ after conventional irradiations. Therefore, we have chosen 25 Gy to perform a first evaluation on whether pMBRT offers a reduction of toxicity over conventional methods at doses high enough to reach a significant probability of glioma sterilization.

For minibeam generation, a multislit brass collimator was employed (400-μm-wide slits, 3200-μm centre-to-centre distance) and was positioned 7 cm away from the rat skin^10^. With this configuration, the minibeam width at a 1.0-cm depth (centre of rat brain) was 1100 ± 50 μm. The peak-to-valley dose ratio at that position was 6.5 ± 0.3. Further details on dosimetry can be found elsewhere^14^.

### Follow-up

The animals were followed-up for 6 months. The health status of each rat was checked once per week. The relative weight compared with that on the day of irradiation was calculated. Rats were euthanized if weight loss exceeded 20%.

An anatomical MRI study was performed at 10 days (n = 3/series), 3 months (n = 3/series), and 6 months (n = 6/series) after irradiation. For each imaging session, a catheter was inserted into the tail vein for contrast agent administration.

A 7-Tesla preclinical magnet (Bruker Avance Horizontal 7-T Bruker, Inc., Billerica, MA) equipped with a 35-mm-diameter “bird-cage” antenna was employed. Three series were acquired:

i) Morphological T2-weighted (T2W) images with a repetition time (TR) of 2500 ms and 4 echo times (TE) of 11, 33, 55 and 77 ms. A signal averaging of 2 was employed. In all, 21 slides were acquired in a total time of 10 min 40 s.
ii) T1-weighted (T1W) TurboRare sequences with a TR of 800 ms and a TE of 6.05 m. A signal averaging of 4 was employed. A total of 21 slides were acquired in a time of 5 min 7 s. Four acquisitions were performed, one before and three (5, 10 and 15 min) after the intravenous injection of a bolus of 100 µmol/kg Gd-DOTA (Guerbet SA, Villepinte, France).
iii) T1 fast low-angle shot (FLASH) sequences with a TR and TE of 114.89 and 3.1 ms, respectively. A flip angle of 30° and a signal averaging of 4 were used. A total of 9 slides were acquired in a total time of 1 min 28 s. Acquisitions were made just before and 5, 10, and 15 min after the intravenous injection of a bolus of 100 µmol/kg Gd-DOTA (Guerbet SA, Villepinte, France).

In all sequences, the field of view was 35 mm × 35 mm, the in-plane resolution amounted to 0.14 mm × 0.14 mm, and the slice thickness and gap were 0.8 and 0.3 mm, respectively.

At the end of the study, the rats were terminally anaesthetised for brain fixation by the intracardiac perfusion of a fixative solution (formalin zinc). The brains were then removed, fixed in the fixative solution, and embedded in paraffin; 4-μm-thick sections were cut and stained in haematoxylin and eosin (HE; detection and general description of histopathological lesion) and Luxol Fast Blue (myelin lesions) for the histopathological (double-blinded) analysis. Immunohistochemistry analysis was performed to assess the networks and cell morphologies of microglia (anti-Iba-1 antibody, Wako Chemicals, dilution: 1:500) and astrocytes (anti-GFAP antibody, Sigma-Aldrich, dilution: 1:500). A quantitative morphometric analysis was carried out to evaluate the density of microglial cells and astrocytes, using ImageJ software version 1.51 (NIH, Bethesda, MD). For each rat, at least 10 areas of brain sections, randomly distributed, were studied at a × 10 magnification. Statistical analysis was performed using GraphPad Prism software (GraphPad, San Diego, CA, USA). One-way analysis of variance (ANOVA) was carried out with a significant level of 0.05. Data are represented as means ± SD; *p < 0.05; **p < 0.01; ***p < 0.001; ****p < 0.0001; no star, statistically not significant.

### Data availability statement

The datasets generated and analyzed during the current study are available from the corresponding author on reasonable request.

## Results

### Animal follow-up

The animals in series 1 (conventional proton irradiation) showed a delay in weight gain in comparison with the unirradiated controls and with the group receiving pMBRT. The maximum weight gain was 15% less in the group treated with conventional proton irradiation than in the two other groups, and those animals started to lose weight from 5 months after irradiation. Moreover, 6 out of 8 rats presented severe clinical symptoms, including apathy and reduced appetite, and 3 rats had to be sacrificed preliminarily due to a weight loss > 20% of their initial weight. All rats irradiated with conventional proton therapy presented permanent epilation and moist desquamation of the irradiated skin area approximately 2 weeks after irradiation. This was treated with Ialuset® PLUS (Laboratories Genevier). In contrast, rats irradiated with pMBRT showed reversible epilation, but only in the path of the minibeams. In series 2 (pMBRT) and series 3 (control), no weight loss was observed throughout the follow-up period. One rat in series 2 died during the MRI procedure performed at 6 months due to the anaesthesia. No clinical symptoms were observed.

**Figure 2:**
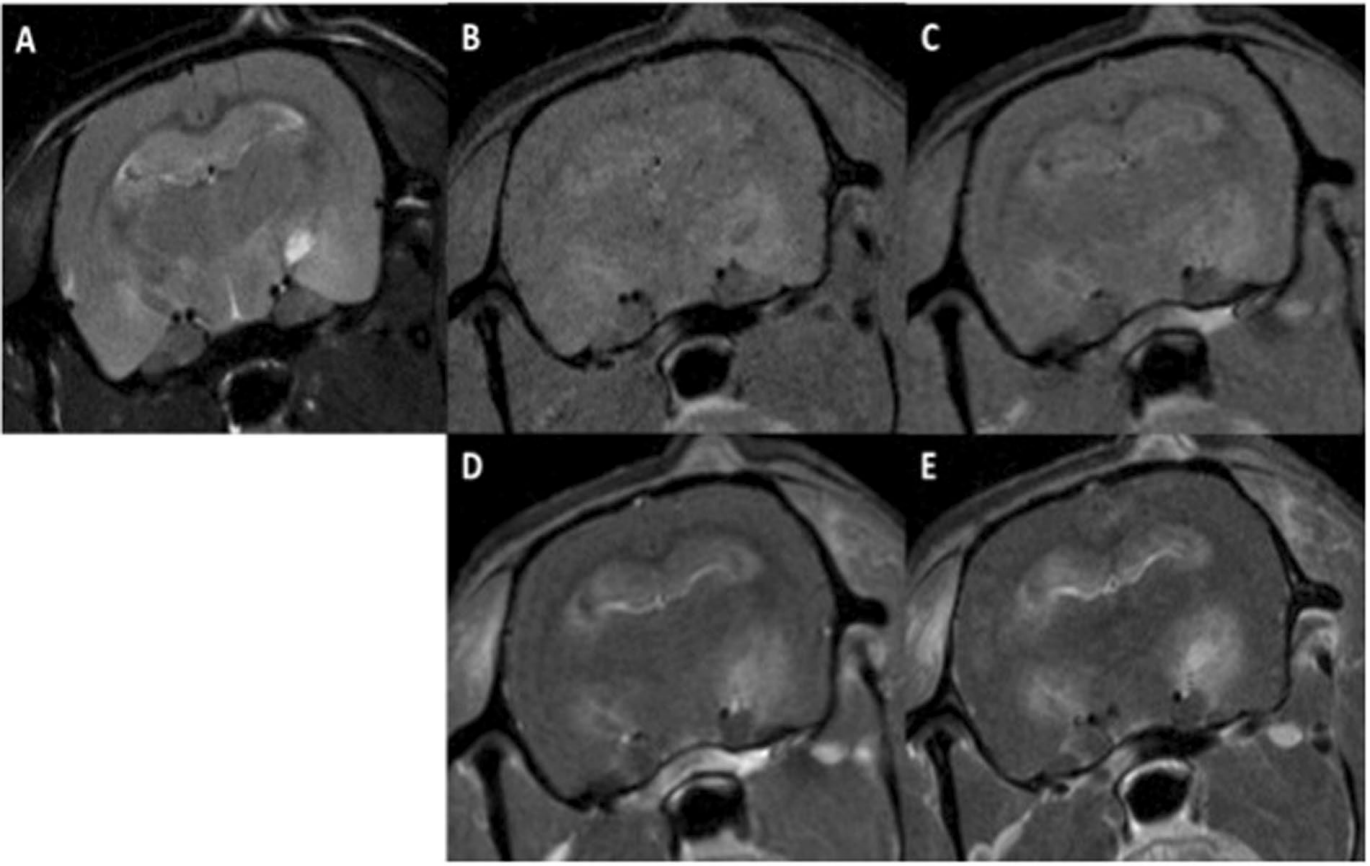
Axial MRI images of one rat in series 1: before Gd injection T2W (**A**), T1W (**B**), and T1 FLASH (**C**), T1W (**D**) and T1 FLASH after Gd injection (**E**). Substantial lesions are present. Some hyperintense lesions in the T2W images, along with high signals in the T1W images and BBB breakdown, are compatible with late acute haemorrhage and can be observed in the hippocampus and subiculum. The hypointense lesion in the T2W images of the left hippocampus, along with high signals in the T1W images indicates early subacute haemorrhage. In the right hippocampus, the bright lesion in the T2W images that appears dark in the T1W images and exhibits high intensity in the T1 FLASH images is compatible with late subacute haemorrhage (methaemoglobin). This lesion touches the ventricular system and one part of the brainstem. Extensive BBB breakdown can be observed in the hippocampal formation along with those areas.

No evident pathologies/lesions were observed in the MRI acquisitions at 10 days and 3 months after irradiation in any of the series. At 6 months after irradiation, MRI revealed severe damage (mainly haemorrhages along with some oedemas) in series 1, which were particularly concentrated in the hippocampal formation (6/6 rats), hypothalamus (4/6 rats) and periaqueductal gray (4/6 rats) areas. Other affected regions were the basal forebrain (2/6 rats) and a small part of the brainstem (2/6 rats). Blood brain barrier (BBB) permeability was observed in all rats imaged in series 1, mainly in the hippocampus (5/6 rats), and/or hypothalamus (5/6 rats). Other areas presenting BBB breakdown were the ventricles (2/6 rats), cortifugal pathways (2/6 rats), basal forebrain (2/6 rats), brainstem (2/6 rats) and septal region (2/6). Figure 2 shows an example of lesions observed.

MRI revealed no significant lesions in series 2.

**Figure 3:**
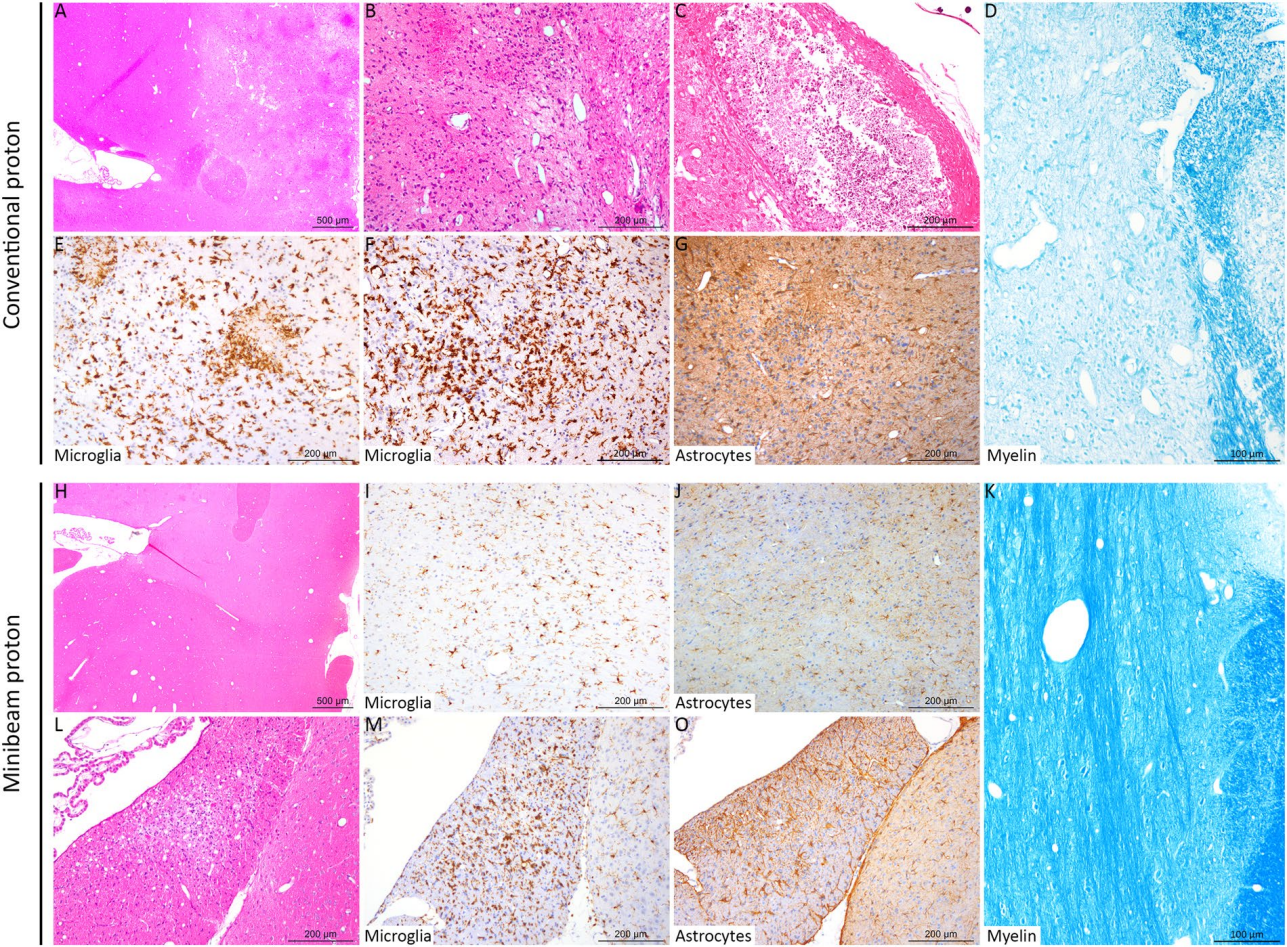
Histopathological and immunohistochemical analyses revealed different lesion profiles between the conventional (**A**–**F**) and minibeam (**G**–**L**) irradiation groups. **A**, **B**, **C**, **H** and **L**: HE staining. **D** and **K**: Luxol Fast Blue staining. E, F, I and M: anti-Iba-1 immunohistochemistry (microglia). **G**, **J** and **O**: anti-GFAP immunohistochemistry (astrocytes). The rats were 32 weeks old when sacrificed. Conventional irradiation: (**A**) Multifocal to coalescing lesion characterized by (**B**) oedema, necrosis and gliosis. (**C**) More severe lesion with cavitation and mineralisation. (**D**) Destruction of the myelin was also observed. (**E**,**F**) Microglial activation and microglial nodules (microgliosis). (**G**) Astrocyte activation with a marked increase in the GFAP immunolabeling (astrogliosis). Minibeam irradiation: (**H**) At low magnification, no lesion was observed in most rats, with (**I**,**J**) normal microglial and astrocytic networks, and (**K**) normal myelin organization. For just one rat: (**L**) One inflammatory infiltrate and mild neuropil destruction was observed, associated with focal (**M**) microgliosis and (**O**) astrogliosis.

**Figure 4:**
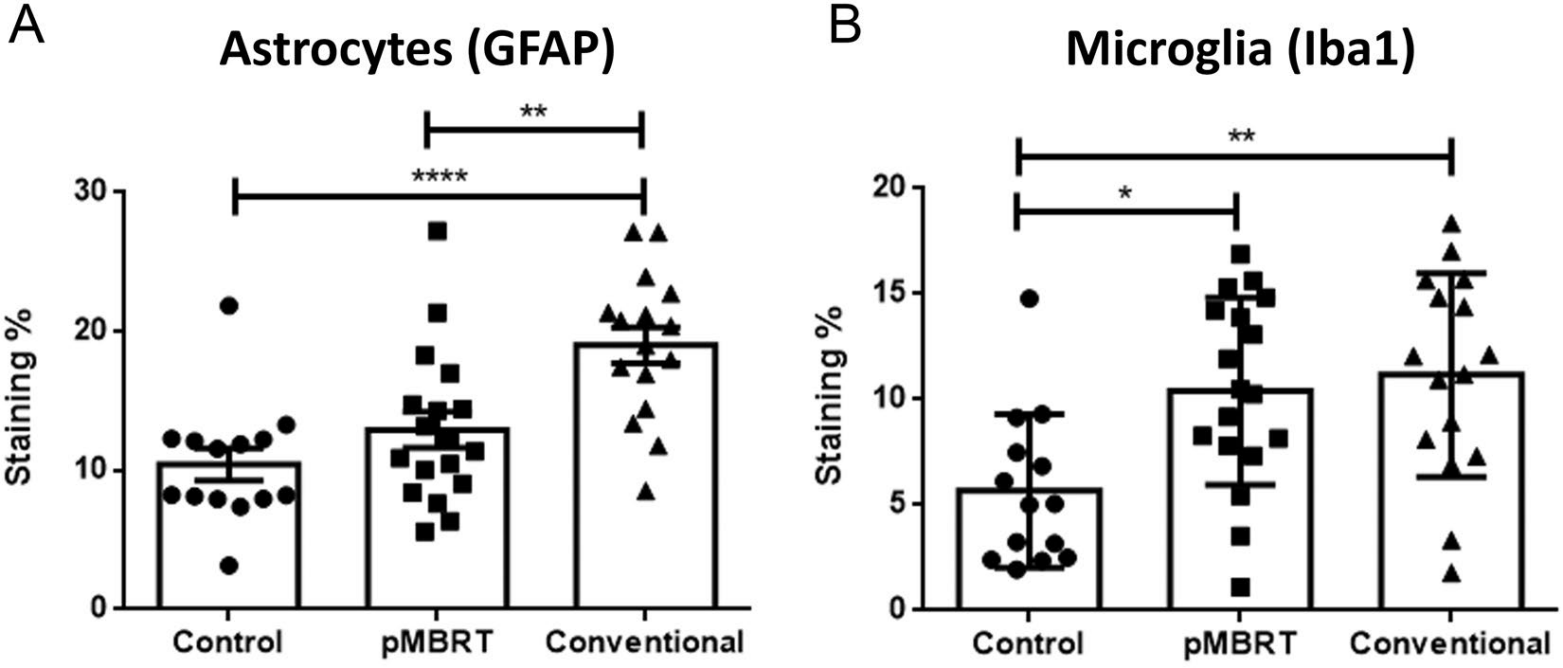
Quantitative morphometric analysis revealed significantly less severe lesions in the pMBRT irradiation group. Activation of astrocytes was significantly stronger in the conventional group (anti-GFAP immunohistochemistry; (**A**) Besides, both pMBRT and conventional treatment provoked activation of microglial cells (anti-Iba1 immunohistochemistry; (**B**) *p < 0.05; **p < 0.01; ***p < 0.001; ****p < 0.0001; no star, statistically not significant.

Histopathological analysis revealed different lesion profiles between the conventional and minibeam irradiation groups. Rats in the conventional group (series 1) displayed multifocal zones of neuropil necrosis and demyelination, sometimes with cavitation and mineralisation (4/8). Immunohistochemical analysis showed multifocal strong activation of microglial cells, *i.e*. neuro-inflammatory processes. We indeed detected an increase in the positive signal after Iba-1 labelling, a thickening of microglial cell body and a thickening and shortening of their cell processes, and the formation of microglial nodules (3/8). After brain insult, astrocytes were also strongly activated with a marked increase of the GFAP positive signal detection. Only one rat in the series did not display any significant histopathological lesions. In contrast, in series 2 (pMBRT), only one out of the eight irradiated animals presented lesions, which were significantly less severe than those observed in the standard proton therapy (PT) group; this animal exhibited only very few microglial nodules (Fig. 3). No large foci of necrosis nor cavitation were observed in any of the rats in this series (Fig. 3). The morphometric analysis (Fig. 4) confirmed these results, revealing (i) the most severe lesions in the conventional irradiation group, (ii) neuro-inflammatory processes with activation of microglial cells in standard PT and pMBRT groups, but significantly less severe activation of astrocytes in pMBRT treated rats. The stronger activation and hyperplasia of astrocytes in the conventional irradiation group is very probably related to the large zones of necrosis and cavitation, forcing a reparation process driven by astrocytes.

## Discussion

In RT, normal tissue tolerances often impose a ceiling on the radiation dose that can be delivered to the tumor, which limits its control. This is especially critical in the case of radio-resistant tumors, such as gliomas. Late radiation effects are the major, dose-limiting complications of brain irradiation. It has been estimated that severe delayed neurotoxicity affects 33% of patients^22^. Injuries include a wide spectrum of abnormalities like inflammation, blood barrier disruption, vascular lesions, demyelination, radio-necrosis and edema^23,24^.

The main goal of the present study was to evaluate whether a significant reduction in neurotoxicity can be attained via the novel strategy we propose, pMBRT, and that at doses high enough to attain a significant probability of glioma sterilization. With that aim, we have compared the side effects of classical homogenous PT and proton minibeam RT irradiations in an *in vivo* animal model (i.e., the rat brain). The comparison was done with clinically relevant beam energies and dimensions, such that the evaluation provides important information for future clinical trials. The same (very high) average dose (25 Gy in one fraction) was delivered to both groups. Along this line, the irradiated animals were followed for 6 months to evaluate long-term effects. Areas of infarction, BBB breakdown and edema were assessed via signal abnormalities in MRI images. Histopathological and immunohistochemical analysis allowed the evaluation of tissue integrity, necrosis, calcifications, and neuroinflammation, among others.

The animals receiving standard PT developed important skin and very severe long-term brain damage, including radionecrosis. In contrast, no skin damage was observed in the pMBRT group, in agreement with the work of Girst *et al*.^16^. In addition, only one out of the eight irradiated rats presented only very few microglial nodules a lesion significantly less severe than those observed in the standard PT group. In addition, the morphometric analysis revealed significantly less severe activation of astrocytes in pMBRT treated rats.

These results indicate the participation of distinct biological mechanisms in pMBRT. Although the pathophysiology of CNS complications of standard RT treatments is still poorly understood, vascular damage is believed to play a central role^23^. In contrast, the hypothesis has emerged in spatially fractionated techniques, such as X-rays microbeam radiation therapy, that the sparing of normal tissues is mainly due to a fast repair of vascular (capillaries) damage^9,11^. In addition to the so-called dose volume-effect^25^, some other effects may be participating. RT can extend its influence beyond what can be attributed to in-field cytotoxicity by modulating the immune system^26^. Whether the amount of inflammation and the response of the immune system in MBRT differ from conventional methods is yet to be explored. In addition, the role of non–targeted effects needs to be disentangled. Another possible player in MBRT, likely linked to cell communication, was hypothesized to be to hyperplasia and migration of endothelium and glial cells from the valleys^2^ to recover the damaged cells in the peaks (high dose areas). All those phenomena challenge many of the current paradigms in conventional radiation therapy, since they seem to implicate different biological mechanisms from those involved when direct damage by ionizing radiation takes place. The recent observations^12^ indicating distinct biological properties of protons, such as complex DNA damage-inducing capacity, could lead to different players acting in X-rays and in proton MBRT. Even if further experiments are needed to unravel the mechanisms taking part in tissue response in pMBRT, our results are a strong indication that this new approach increases normal brain resistance to radiation. Here we present a remarkable tissue-sparing effect of 57- Gy supra-millimetre minibeams (1.1 mm at 1-cm depth). It is a valuable finding in the context of general indication in the literature that the minibeams’ tissue-sparing effect starts to rapidly decline above 0.7 mm beam thickness when used with much higher x-ray minibeam doses^13^. These results have important implications for potential clinical trials, since it opens the door for future implementations with pencil beam scanning systems (millimetre-size beams). In addition, to the best of our knowledge this is the first time that the effects of such an irradiation (whole brain) with an array of supra-millimetric beams have been evaluated.

Since a homogeneous dose distribution can be obtained in the target via pMBRT^1^, these results can be directly translated either to an important reduction in the probabilities of normal tissue complications for the same tumour control probability (same dose in the tumour) or to the deposition of greater and potentially curative doses in the case of radio-resistant tumours, in particular, gliomas. This approach might also allow retreatment of the brain months or years after the initial radiation treatment(s). In addition, it can specially benefit paediatric oncology (involving the central nervous system), whose treatments are very limited due to the risk of complications in the child’s development.

## Conclusions

The results from our animal study provide clear evidence of the shift to higher doses of the normal tissue complication probability curve in pMBRT in the brain. This net gain in normal tissue sparing can foster one of the main applications of proton therapy, paediatric oncology, as well as open the door to the efficient treatment of very radio-resistant tumours, such as gliomas, which are currently mostly treated palliatively.
